# Estimated Prevalence of Depressive Disorders in Children From 2004 to 2019: A Systematic Review and Meta-Analysis

**DOI:** 10.1001/jamapediatrics.2023.3221

**Published:** 2023-08-28

**Authors:** Michael J. Spoelma, Gemma L. Sicouri, Deanna A. Francis, Annabel D. Songco, Emily K. Daniel, Jennifer L. Hudson

**Affiliations:** 1 Black Dog Institute, University of New South Wales, Sydney, Australia; 2 Discipline of Psychiatry and Mental Health, School of Clinical Medicine, Faculty of Medicine and Health, University of New South Wales, Sydney, Australia; 3 School of Psychology, Faculty of Science, University of New South Wales, Sydney, Australia; 4 Faculty of Medicine and Health, University of New South Wales, Sydney, Australia

## Abstract

**Importance:**

Depression during childhood (ie, age <13 years) poses a major health burden. Recent changes in environmental and lifestyle factors may increase children’s risk of mental health problems. This has been reported for anxiety disorders, but it is unclear whether this occurs for depressive disorders.

**Objective:**

To provide prevalence estimates for the depressive disorders (ie, major depressive disorder [MDD], dysthymia, disruptive mood dysregulation disorder [DMDD], and overall) in children, and whether they have changed over time.

**Data Sources:**

The MEDLINE, PsycINFO, Embase, Scopus, and Web of Science databases were searched using terms related to depressive disorders, children, and prevalence. This was supplemented by a systematic gray literature search.

**Study Selection:**

Studies were required to provide population prevalence estimates of depressive disorder diagnoses (according to an established taxonomy and standardized interviews) for children younger than 13 years, information about participants’ year of birth, and be published in English.

**Data Extraction and Synthesis:**

Data extraction was compliant with the Meta-Analysis of Observational Studies in Epidemiology guidelines. A total of 12 985 nonduplicate records were retrieved, and 154 full texts were reviewed. Data were analyzed from 2004 (the upper limit of a previous review) to May 27, 2023. Multiple proportional random-effects meta-analytic and mixed-effects meta-regression models were fit.

**Main Outcomes and Measures:**

Pooled prevalence rates of depressive disorders, prevalence rate differences between males vs females and high-income countries (HICs) vs low-and middle-income countries (LMICs), and moderating effects of time or birth cohort.

**Results:**

A total of 41 studies were found to meet the inclusion criteria. Pooled prevalence estimates were obtained for 1.07% (95% CI, 0.62%-1.63%) for depressive disorders overall, 0.71% (95% CI, 0.48%-0.99%) for MDD, 0.30% (95% CI, 0.08%-0.62%) for dysthymia, and 1.60% (95% CI, 0.28%-3.90%) for DMDD. The meta-regressions found no significant evidence of an association with birth cohort, and prevalence rates did not differ significantly between males and females or between HICs and LMICs. There was a low risk of bias overall, except for DMDD, which was hindered by a lack of studies.

**Conclusions and Relevance:**

In this systematic review and meta-analysis, depression in children was uncommon and did not increase substantially between 2004 and 2019. Future epidemiologic studies using standardized interviews will be necessary to determine whether this trend will continue into and beyond the COVID-19 pandemic.

## Key Points

**Question:**

What were the prevalence rates of depressive disorders in childhood (ie, age <13 years) between 2004 and 2019?

**Findings:**

In this systematic review and meta-analyses of 41 studies, pooled prevalence estimates were noted for major depressive disorder (0.71%), dysthymia (0.30%), disruptive mood dysregulation disorder (1.60%), and 1.07% overall. These estimates did not differ significantly between males and females or high-income and low- and-middle-income countries and did not appear to increase over a 15-year period.

**Meaning:**

These findings suggest that depression in childhood between 2004 and 2019 was uncommon and did not increase over time, but the lack of data beyond the COVID-19 pandemic is yet to be accounted for.

## Teaser

This meta-analysis examines the prevalence of major depressive disorder, dysthymia, and disruptive mood dysregulation disorder in children.

##  Introduction

Depression is one of the most burdensome and leading causes of disability worldwide.^1^ In children, depression can occur in those aged as young as 3 years,^2^ but this was largely unrecognized until the 1980s, with the publication of the third edition of the *Diagnostic and Statistical Manual of Mental Disorders* (*DSM-III*). Depression during childhood is uncommon but is nevertheless an important clinical priority given the greater severity of long-term mental health sequelae compared with individuals with a later age of onset.^3,4,5^ This rarity could also explain why many clinicians report lacking confidence in treating depression in children, further exacerbated by receiving inadequate training.^6^ As such, compared with adolescents, there are lower rates of children receiving appropriate evidence-based care for depression,^7^ despite there being a strong evidence base for interventions such as cognitive behavioral therapy.^8,9^

There have been noticeable environmental changes to childhood experiences over the last 2 decades that may have negatively affected child mental health outcomes. Family dynamics, for example, have changed in that there is greater parental involvement in children’s activities than in the past.^10^ This could potentially lead parents into adopting a more overinvolved parenting style, which has been linked to increased depressive symptoms.^11^ Various sedentary behaviors have also become more prevalent. There has also been an increase in screen time: as many as two-thirds of children are estimated to exceed recommended limits.^12^ In turn, this affects sleep,^13^ physical activity levels,^14^ and diet,^15^ which all exist in a bidirectional relationship with depression where one exacerbates the other.^16,17,18,19^ Furthermore, weight issues are of particular importance, since there is evidence to suggest that increasing obesity rates have resulted in earlier puberty onset.^20,21^ Such a shift is substantial, given that puberty-related biological processes have been reported to affect developmental factors, which results in an increased risk of anxiety and depressive disorders.^22^ In addition, links between early puberty and greater mental health problems have been observed.^23^

In the last 2 decades, there has been an increase in the prevalence of anxiety disorders in children (1 estimate quantified an increase from 3.5% in 2003 to 4.1% in 2011-2012).^24^ This raises concerns as to whether a similar increase is occurring for depression, given the high comorbidity between the conditions.^25,26,27,28,29^ Currently, the evidence is unclear.^24,30^ However, these trends are more often observed in combined child and adolescent populations, and the outcomes in children 12 years and under specifically are unknown. Many epidemiologic studies have been conducted to determine the prevalence of depression in childhood, but there have been few meta-analyses, and even fewer that have considered prevalence changes over time.

One exception is a meta-analysis by Costello et al,^31^ which examined multiple studies from 1987 to 2004 and obtained an overall prevalence rate of 2.8% (SE, 0.5%) for depressive disorders in children younger than 13 years. They also found that, contrary to reported concerns of an epidemic of depression in childhood, prevalence rates had not increased over time. They suggested the epidemic observation likely reflected greater clinician awareness that has rectified historical rates of underdiagnosis. However, a few caveats to this result warrant further study. First, they did not offer separate estimates for specific depressive disorders, such as major depressive disorder (MDD), dysthymia (DYS), or disruptive mood dysregulation disorder (DMDD) (first introduced in *Diagnostic and Statistical Manual of Mental Disorders, Fifth Edition* [*DSM-5*]). Additionally, these estimates only report studies up to 2004, and the current outlook may be considerably different.

A recent increase in childhood depressive disorder prevalence might therefore be plausible, but previous research has not yet assessed this directly. The aim of this study was to update and extend the results of Costello et al^31^ and provide prevalence estimates of depressive disorders in children (as defined by established taxonomies, namely the *DSM* and *International Statistical Classification of Diseases and Related Health Problems, 10th Revision* [*ICD-10*]) and how this has changed over time.

## Methods

In addition to the information given herein, the eMethods in Supplement 1 provides further details on the study method and its justification, in addition to the gray literature search strategy, analytic procedures, quality assessments, and definitions of the depressive disorders. This study followed the Meta-Analysis of Observational Studies in Epidemiology (MOOSE) reporting guideline.^32^

### Search Strategy and Study Selection

A literature search was conducted in MEDLINE, PsycINFO, Embase, Scopus, and Web of Science for studies conducted from 2004 (the upper limit of the Costello et al^31^ search) to May 27, 2023. The search terms are provided in eTable 1 in Supplement 1. Additional searches of the gray literature were also conducted independently by 2 of us (M.J.S. and E.K.D.). Briefly, the first 100 results of a Google search of *child depressive disorder prevalence* were used to identify potentially relevant stakeholder organizations. The national statistical agencies and government health departments/agencies of major Anglophonic countries (Australia, New Zealand, UK, US, Canada, and Ireland) were also added to this list. All Google results were screened, and the organization websites were searched either manually or through the websites’ search functions for studies and/or relevant data. eTable 2 in Supplement 1 lists the organizations considered in this search.

Inclusion criteria were similar to those of Costello et al.^31^ Specifically, studies were required to (1) provide prevalence estimates for individuals younger than under 13 years (range, 0-12 years), (2) have defined depressive disorder diagnoses based on an established taxonomy (ie, any version of *DSM*, *ICD*) and standardized structured/semistructured interview, and (3) have ascertainable information about the birth cohort of the participants. As additional requirements, all studies had to (4) produce population data (ie, not a clinical sample or restricted to a particular social group), (5) be published in English, (6) separate depressive disorders from other mood/affective disorders (eg, bipolar disorder), and (7) started/finished data collection no earlier/later than 2002/2019 (eMethods in Supplement 1 provides justification for this).

**Figure 1.**
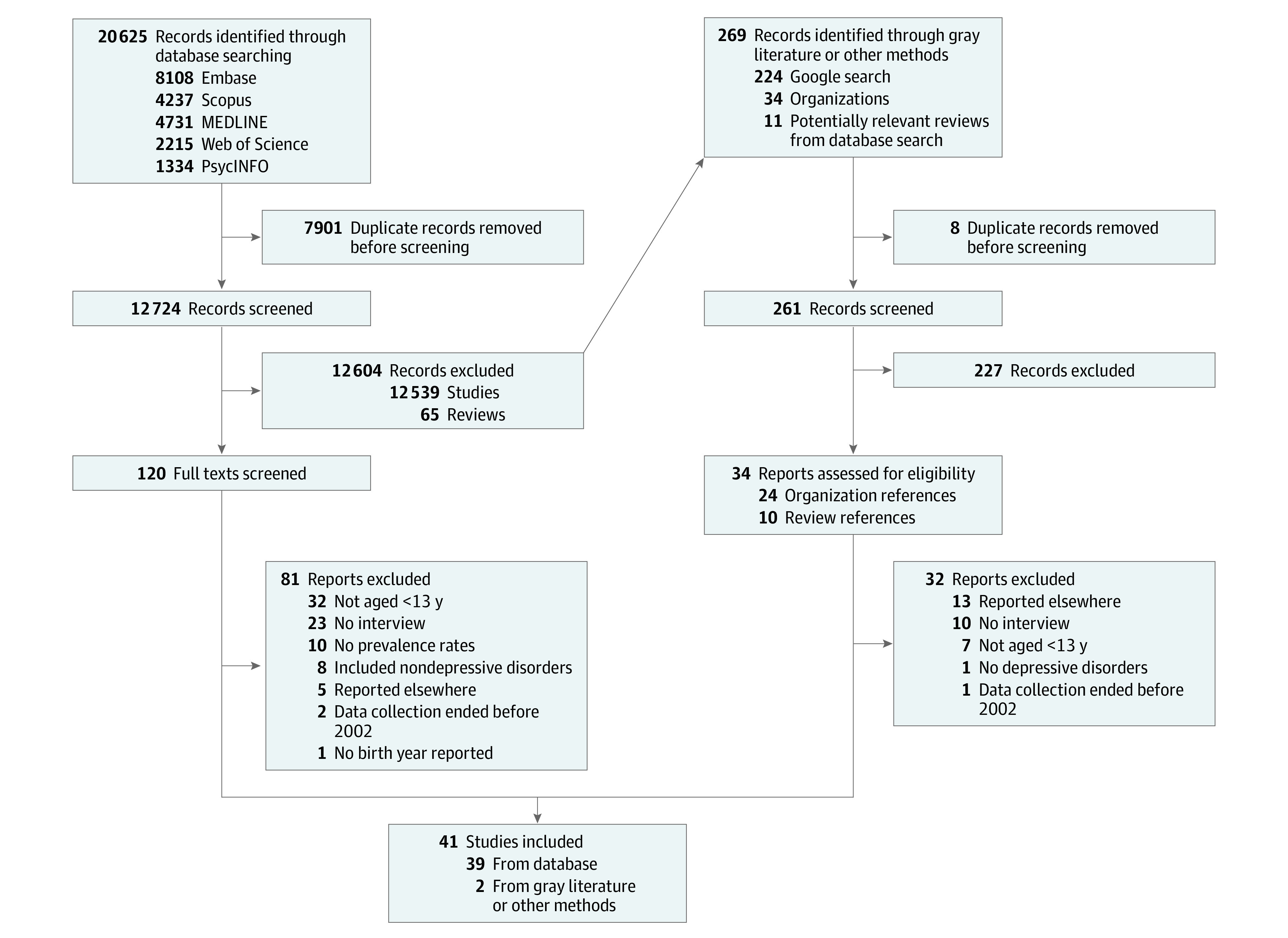
Preferred Reporting Items for Systematic Reviews and Meta-Analyses Flow Diagram Outlining the Search Strategy and Study Selection Process

Study selection was independently performed by one of us (M.J.S.) and another author (either G.L.S., A.D.S., E.K.D., or J.L.H.), using EndNote 20 and Covidence, and followed a 2-stage screening process. The titles and abstracts of all articles were screened, then potentially relevant articles had their full texts reviewed. At both stages, any conflicts were resolved through discussion. Potentially relevant review articles were also identified during this first stage, and their reference lists were checked for further studies. This process is illustrated in Figure 1.

### Data Extraction

Once studies were selected, 2 of us (M.J.S. and J.L.H.) extracted the following data: the country the study was conducted in, the taxonomy and interviews used to determine diagnoses, the interview informants, the depressive diagnoses, the participants’ year of birth and age range, the sample size, and the diagnostic prevalence rates (we were interested in 3 depressive disorders: MDD, DYS, and DMDD, in addition to an estimate for depressive disorders overall [ALL]). If available, the latter 2 features were also extracted separately for the sexes (assigned at birth, ie, male and female).

### Data Synthesis and Analysis

The main analyses consisted of random effects (to account for expected study heterogeneity) proportional meta-analytic models. If a study provided both *DSM*- and *ICD*-based prevalence estimates, the *DSM* estimates were used given their greater popularity in our included studies. Further models were also fit considering only males or females for studies that offered separate estimates for each sex. If relevant and appropriate, prevalence estimates between the models were compared using 2-sample *z* tests.

The models were fit using restricted maximum likelihood estimators and Freeman-Tukey double-arcsine transformed estimates with Hartung-Knapp-Sidik-Jonkman CIs. If the number of studies included in any of the analyses was 10 or larger, mixed-effects meta-regressions were conducted to ascertain the association of a few key moderators with prevalence rates. We were particularly interested in the association between birth cohort and prevalence rates and whether this indicated an increase in depressive disorder prevalence over time. However, to account for plausible confounders, the models also considered the median age of the participants and the time frame of the interview. Taxonomy and interview types have previously been considered by Costello et al^31^; however, we opted against including these in the models. This was due to the lack of appreciable differences in diagnostic criteria for more recent versions of the *DSM* and the number of different interviews that were conducted. Finally, to consider the generalizability of our estimates, we also compared low- and middle-income countries (LMICs) with high-income countries (HICs); eMethods in Supplement 1 provides definitions. This was particularly relevant given that only one study analyzed by Costello et al^31^ was from an LMIC.

Study heterogeneity in both the meta-analyses and meta-regressions were assessed by the calculation of prediction intervals and leave-1-out sensitivity analyses. Bias and study quality were assessed for each study by 2 of us (M.J.S. and A.D.S. or E.K.D.) using the Joanna Briggs Institute Critical Appraisal Checklist for Prevalence Studies (JBIC). The JBIC produces a numeric score indicating how many of its 9 items were fulfilled; the eMethods in Supplement 1 provides further information.^33^ Our confidence in the overall study quality and prevalence estimates was assessed using the Grading of Recommendation, Assessment, Development, and Evaluation (GRADE) approach.^34^ All analyses were conducted in R, version 4.3.0 and included meta-analytic models fit using the *metafor* package (R Foundation for Statistical Computing).^35^ All significance tests and statistics were unpaired, 2-sided, and had a statistical significance threshold of *P* < .05.

## Results

### Study Characteristics

**Figure 2.**
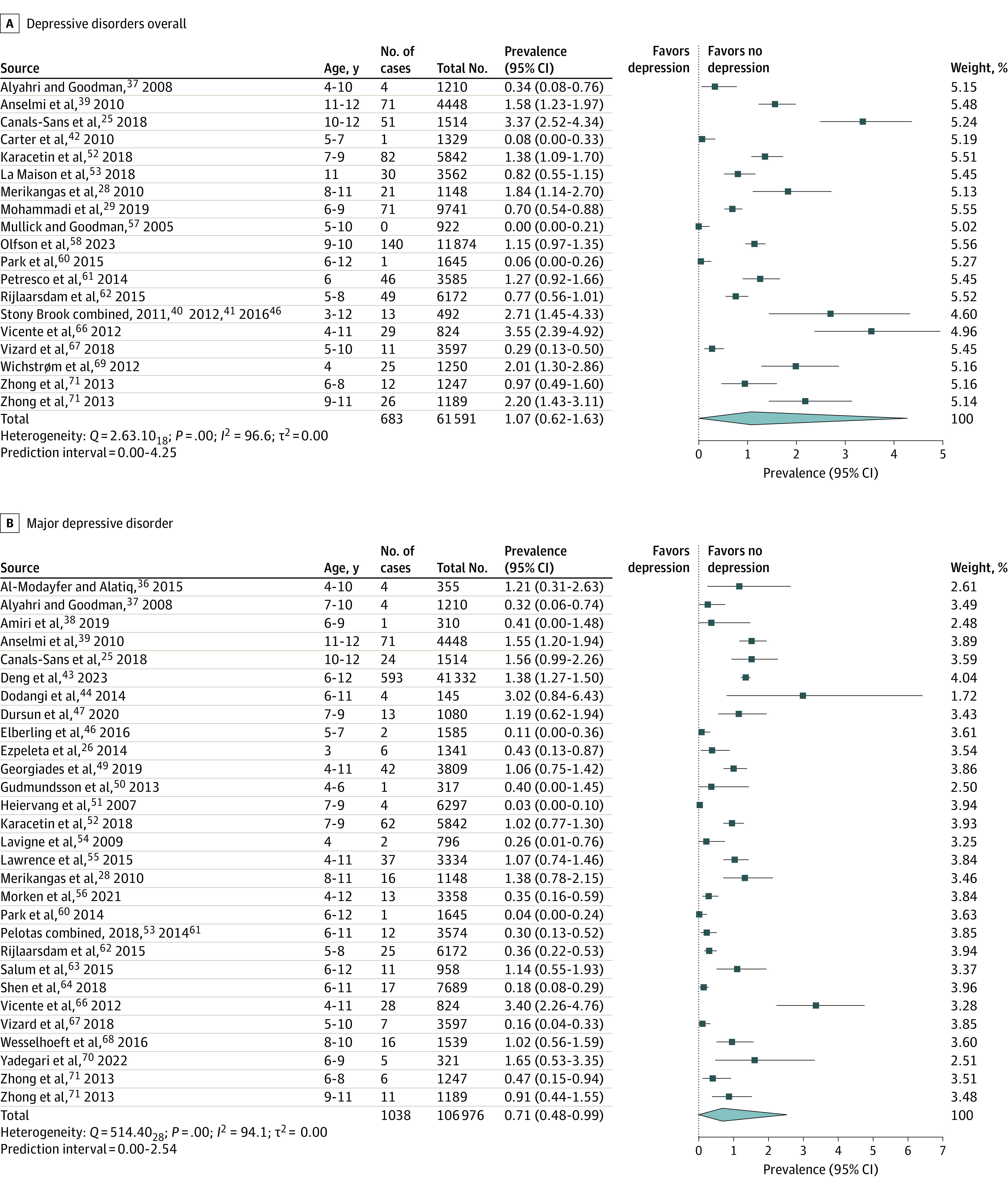
Meta-Analyses of Depressive Disorders Overall and Major Depressive Disorder

**Figure 3.**
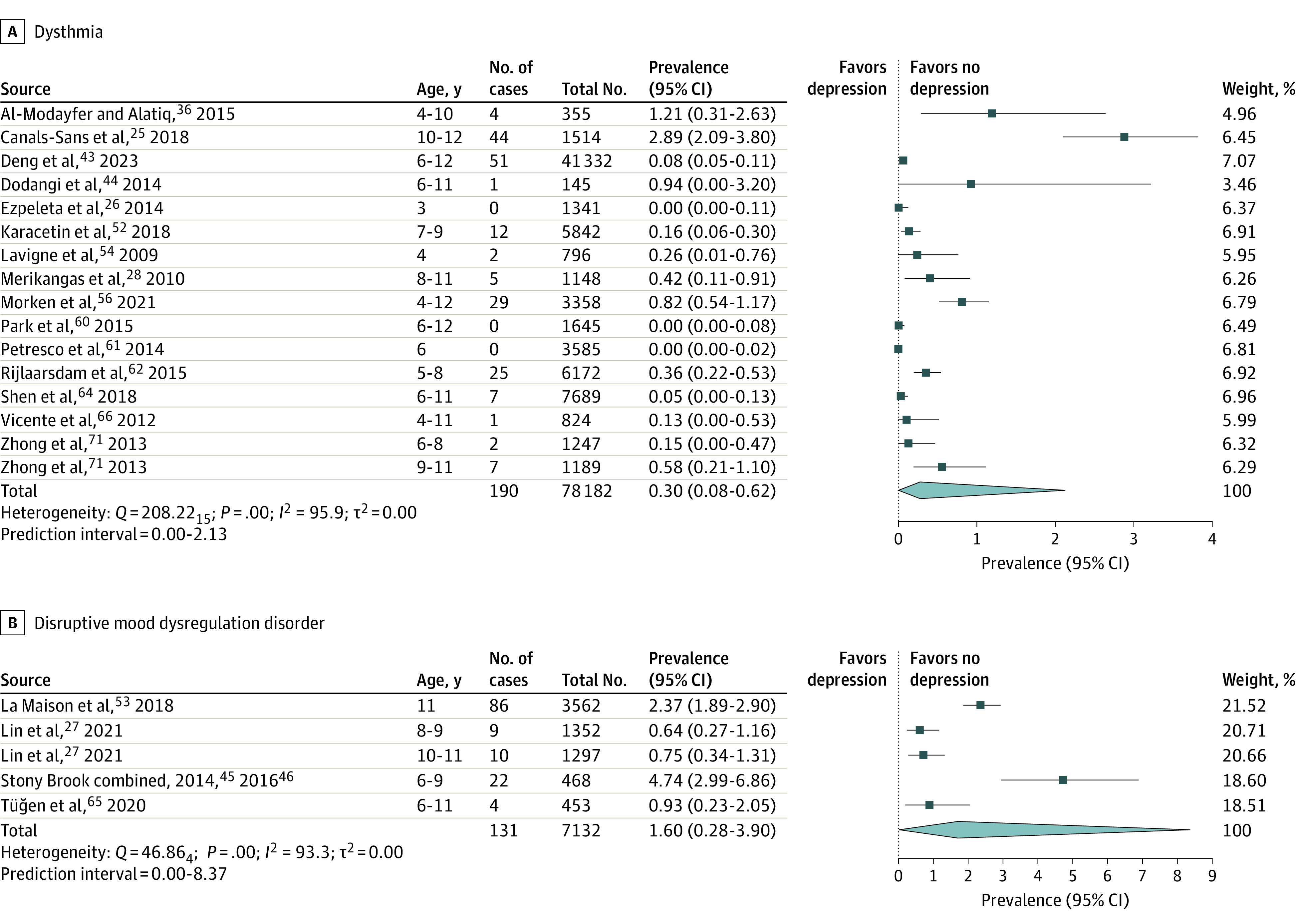
Meta-Analyses of Dysthymia and Disruptive Mood Dysregulation Disorder

A total of 12 985 nonduplicate records were retrieved, and 154 full texts were reviewed. In total, 41 studies were identified as meeting study criteria.^25,26,27,28,29,36,37,38,39,40,41,42,43,44,45,46,47,48,49,50,51,52,53,54,55,56,57,58,59,60,61,62,63,64,65,66,67,68,69,70,71^ Table 1 lists the characteristics of all studies included in at least 1 of the meta-analyses, while the sample size and prevalence rates are reported in the forest plots shown in Figure 2 and Figure 3. Of these 41 studies, a prevalence estimate for ALL could be ascertained for 19 cohorts. More diagnosis-specific estimates were provided by 29 cohorts for MDD, 16 for DYS, and 5 for DMDD. Further study characteristics relevant to the preprocessing of data for analyses are discussed in the eResults in Supplement 1.

**Table 1.** Characteristics of the Studies Included in the Meta-Analyses

Source	Country	Taxonomy	Interview	Informants	Time frame, mo	Diagnoses	Year of birth	Age at interview, y
Al-Modayfer and Alatiq,^36^ 2015	Saudi Arabia	*DSM-IV*	MINI-K	Parent/guardian	Current	MDD, DYS	2001-2008	4-10
Alyahri and Goodman,^37^ 2008	Yemen	*DSM-IV*	DAWBA	Parent/guardian	Current	MDD, ALL	1992-1996	7-10
Amiri et al,^38^ 2019	Iran	*DSM-IV*	K-SADS-PL	Parent/guardian, child	Current	MDD	2007-2011	6-9
Anselmi et al,^39^ 2010	Brazil	*DSM-IV*,* ICD-10*	DAWBA	Parent/guardian, child	Current	MDD, ALL	1993	11-12
Bufferd et al,^40^ 2011	US	*DSM-IV*	PAPA	Parent/guardian	3	ALL	2001-2004	3
Bufferd et al,^41^ 2012	US	*DSM-IV*	PAPA	Parent/guardian	3	ALL	2001-2004	6
Canals-Sans et al,^25^ 2018	Spain	*DSM-5*	MINI-K	Child	Current	MDD, DYS, ALL	1995-1998	10-12
Carter et al,^42^ 2010	USA	*DSM-IV*	DISC-IV	Parent/guardian	1	MDD	1995-1997	5-7
Deng et al,^43^ 2023	China	*DSM-IV*, *ICD-10*	MINI-K	Parent/guardian	Current	MDD, DYS	2001-2009	6-12
Dodangi et al,^44^ 2014	Iran	*DSM-IV-TR*	K-SADS-PL	Parent/guardian, child	Current	MDD, DYS	2000-2007	6-11
Dougherty et al,^45^ 2014	USA	*DSM-5*	PAPA	Parent/guardian	3	DMDD	2001-2004	6
Dougherty et al,^46^ 2016	USA	*DSM-5*	K-SADS-PL	Parent/guardian, child	Current	DMDD, ALL	2001-2004	9
Dursun et al,^47^ 2020	Türkiye	*DSM-IV*	DAWBA	Parent/guardian	Current	MDD	2005-2008	7-9
Elberling et al,^48^ 2016	Denmark	*ICD-10*	DAWBA	Parent/guardian	Current	MDD	2000	5-7
Ezpeleta et al,^26^ 2014	Spain	*DSM-IV*	DICA-PPC	Parent/guardian	Current	MDD, DYS	2005-2007	3
Georgiades et al,^49^ 2019	Canada	*DSM-IV-TR*	MINI-K	Parent/guardian	6	MDD	2002-2010	4-11
Gudmundsson et al,^50^ 2013	Iceland	*DSM-IV*	K-SADS-PL	Parent/guardian	Current	MDD	1997-1999	4-6
Heiervang et al,^51^ 2007	Norway	*DSM-IV*	DAWBA	Parent/guardian	Curr	MDD	1993-1995	7-9
Karacetin et al,^52^ 2018	Türkiye	*DSM-IV*	K-SADS-PL	Parent/guardian,	Current	MDD, DYS, ALL	2005-2008	7-9
La Maison et al,^53^ 2018	Brazil	*DSM-5*, *ICD-10*	DAWBA	Parent/guardian	Current	DMDD, MDD, ALL	2004	11
Lavigne et al,^54^ 2009	US	*DSM-IV*	DISC-YC	Parent/guardian	3 (MDD); 12 (DYS)	MDD, DYS	2002-2004	4
Lawrence et al,^55^ 2015	Australia	*DSM-IV*	DISC-IV	Parent/guardian, child	12	MDD	2001-2010	4-11
Lin et al,^27^ 2021	Taiwan	*DSM-5*	K-SADS-E	Child	Current	DMDD	2004-2008	8-11
Merikangas et al,^28^ 2010	USA	*DSM-IV*	DISC-IV	Parent/guardian	12	MDD, DYS, ALL	1989-1996	8-11
Mohammadi et al,^29^ 2019	Iran	*DSM-IV-TR*	K-SADS-PL	Parent/guardian, child	Current	ALL	2006-2011	6-9
Morken et al,^56^ 2021	Norway	*DSM-5*	PAPA (4-6) CAPA (8-12)	Parent/guardian, child	3	MDD, DYS	2003-2004	4-12
Mullick and Goodman,^57^ 2005	Bangladesh	*ICD-10*	DAWBA	Parent/guardian, teacher	Current	ALL	1993-1999	5-10
Munhoz et al,^58^ 2017	Brazil	*DSM-5*	DAWBA	Parent/guardian	Current	DMDD	2004	11
Olfson et al,^59^ 2023	USA	*DSM-5*	K-SADS-C	Parent/guardian, child	Current	ALL	2005-2009	9-10
Park et al,^60^ 2015	South Korea	*DSM-IV*	DISC-IV	Parent/guardian	12	MDD, DYS, ALL	1992-2000	6-12
Petresco et al,^61^ 2014	Brazil	*DSM-IV, ICD-10*	DAWBA	Parent/guardian	Current	MDD, DYS, ALL	2004	6
Rijlaarsdam et al,^62^ 2015	Netherlands	*DSM-IV*	DISC-YC	Parent/guardian	3	MDD, DYS, ALL	2002-2006	5-8
Salum et al,^63^ 2015	Brazil	*DSM-IV*	DAWBA	Parent/guardian	Current	MDD	1997-2004	6-12
Shen et al,^64^ 2018	China	*DSM-IV*	MINI-K	Parent/guardian, child	12	MDD, DYS	2004-2011	6-11
Tüğen et al,^65^ 2020	Türkiye	*DSM-5*	K-SADS-PL	Child	Current	DMDD	2006-2012	6-11
Vicente et al,^66^ 2012	Chile	*DSM-IV*	DISC-IV	Parent/guardian, child	12	MDD, DYS, ALL	1995-2005	4-11
Vizard et al,^67^ 2018	England	*ICD-10*	DAWBA	Parent/guardian, teacher	Current	MDD, ALL	2006-2012	5-10
Wesselhoeft et al,^68^ 2016	Denmark	*DSM-IV*	DAWBA	Parent/guardian	Current	MDD	2000-2003	8-10
Wichstrøm et al,^69^ 2012	Norway	*DSM-IV*	PAPA	Parent/guardian	3	MDD, DYS, ALL	2003-2004	4
Yadegari et al,^70^ 2022	Iran	*DSM-IV*	K-SADS-PL	Parent/guardian	Current	MDD	2006-2011	6-9
Zhong et al,^71^ 2013	China	*DSM-IV*	MINI-K	Parent/guardian, child	Current	MDD, DYS, ALL	1998-2004	6-11

Abbreviations: ALL, All depressive disorders; CAPA, Child and Adolescent Psychiatric Assessment; DAWBA, Development and Well-Being Assessment for Children and Adolescents; DICA-PPC, Diagnostic Interview of Children and Adolescents for Parents of Preschool Children; DISC-IV, National Institute of Mental Health Diagnostic Interview Schedule for Children Version-IV; DISC-YC, Diagnostic Interview Schedule for Children–Young Child; DMDD, disruptive mood dysregulation disorder; *DSM-IV*, *Diagnostic and Statistical Manual of Mental Disorders; Fourth Edition*; *DSM-IV-TR*, *Diagnostic and Statistical Manual of Mental Disorders; Fourth Edition, Text Revision*; *DSM-5*, *Diagnostic and Statistical Manual of Mental Disorders, Fifth Edition*; DYS, dysthymia; *ICD-10*, *International Statistical Classification of Diseases and Related Health Problems, 10th Revision*; K-SADS-C, Kiddie Schedule for Affective Disorders and Schizophrenia–Computerized Version; K-SADS-E, Kiddie Schedule for Affective Disorders and Schizophrenia for School-Aged Children–Epidemiologic; K-SADS-PL, Kiddie Schedule for Affective Disorders and Schizophrenia for School-Aged Children Present and Lifetime; MDD, major depressive disorder; MINI-K, Mini-International Neuropsychiatric Interview for Kids; PAPA, Preschool Age Psychiatric Assessment.

### Prevalence Estimates

Figure 2 and Figure 3 show the forest plots associated with each of the 4 primary meta-analyses. The pooled prevalence estimates obtained were 1.07% (95% CI, 0.62%-1.63%) for depressive disorders overall, 0.71% (95% CI, 0.48%-0.99%) for MDD, 0.30% (95% CI, 0.08%-0.62%) for DYS, and 1.60% (95% CI, 0.28%-3.90%) for DMDD. Our overall depression prevalence estimate was significantly lower (less than half) than the 2.8% figure (0.5%) obtained by Costello et al^31^ (difference *z* = 3.08; *P* = .002).

Sensitivity analyses revealed that excluding individual studies resulted in prevalence changes of no more than 0.10% for the ALL analysis, 0.06% for MDD, 0.10% for DYS, and 0.47% for DMDD. Prediction intervals (reported in Figure 2 and Figure 3) were also quite small, with plausible ranges not exceeding a maximum of 4.25% for the ALL analyses, 2.54% for MDD, 2.13% for DYS, and 8.37% for DMDD.

### Subgroup Analyses and Meta-Regressions

Table 2 displays the results of subgroup analyses examining differences between HICs and LMICs and males and females. For the former, HICs comprised 10 of the studies and LMICs 9 of the studies for the ALL analyses, 16 and 13 studies for MDD, and 9 and 7 studies for DYS. Multiple *z* tests revealed that there were no significant differences in disorder prevalence between HICs and LMICs for any of the analyses. Similar results were found for males vs females, for which sex-separated estimates were provided by 10 studies for the ALL analyses, 9 for MDD, and 5 for DYS. Only 1 study^58^ provided separate DMDD estimates for males and females, which quantified a prevalence rate of 2.88% (n = 1804) for males and 2.02% (n = 1686) for females.

**Table 2.** Results of the Subgroup Analyses and Meta-Regression Models^a^

Model	ALL			MDD			DYS		
	Value	SE	*P* value	Value	SE	*P* value	Value	SE	*P* value
Subgroup analyses, prevalence, %									
Male vs female									
Male	0.85	0.38	.84	0.66	0.26	.60	0.26	0.42	.87
Female	0.74	0.35		0.49	0.19		0.38	0.56	
HICs v LMICs									
HICs	1.24	0.49	.58	0.63	0.19	.45	0.45	0.28	.25
LMICs	0.92	0.29		0.83	0.19		0.11	0.09	
Meta-regression,^b^ β (95% CI)									
Age	0.005735 (−0.008036 to 0.019506)	0.006461	NA	0.009012 (0.000407 to 0.017617)	0.004178	NA	0.009732 (−0.000761 to 0.020225)	0.004816	NA
Birth year	0.002065 (−0.003616 to 0.007745)	0.002665	NA	0.001464 (−0.001723 to 0.004651)	0.001547	NA	−0.002442 (−0.008186 to 0.003302)	0.002636	NA
Interview time	0.002715 (−0.004399 to 0.009828)	0.003337	NA	0.000618 (−0.002822 to 0.004058)	0.001670	NA	−0.004316 (−0.008902 to 0.000269)	0.002105	NA

Abbreviations: ALL, All depressive disorders; DYS, dysthymia; HICs, high-income countries; LMICs, low- and-middle-income countries; MDD, major depressive disorder; NA, not applicable.

^a^ All continuous covariates were mean centered. For age and birth year, covariate values were taken to be the midpoint of the range for each study. Coefficients corresponded to the change given a 1-year increase for age, birth year, and interview time.

^b^ Results reported here are based on the Freeman-Tukey double arcsine transformed estimates. Numbers cited in the article body were back-transformed.

The meta-regression models outlined previously were conducted for the ALL, MDD, and DYS analyses and are reported in Table 2. Birth year was not associated with a change in prevalence for any analysis, and there was no significant difference in interview time frame for the ALL and MDD analyses. However, age was associated with MDD prevalence; a 1-year increase in age corresponded to a prevalence increase of 0.16% for MDD. There was also a nonsignificant increase of 0.16% for DYS. The DYS analyses also suggested that examining prevalence over a longer period decreased the prevalence by approximately 0.08% for each extra month.

### Study Bias and Quality Assessment

eTable 3 in Supplement 1 lists the bias assessments for the 41 studies according to the JBIC. The median total score was 6/9. The standardized interview inclusion criterion automatically meant that the method used for depression identification was acceptable. Other strengths of the studies were evident in their sampling procedures, both in terms of sampling frames and sampling strategies (both 38 of 41 [92.68%]). More common weaknesses included insufficient accounting for (or failure to report) low response rates/dropouts (16 of 41 [39.02%]), the reliability of standardized interviews across participants (16 of 41; [39.02%]), and differences in subgroup response rates (17 of 41 [41.46%]).

The GRADE assessments are provided in eTable 4 in Supplement 1. Overall, we evaluated the evidence overall as being of high quality. While the JBIC results suggest serious issues for some studies, their contribution to the overall estimates is minimal enough to not affect the stability of the estimates to a notable degree. However, we would consider the DMDD evidence to be low quality given the limited number of studies and wide variety of estimates.

## Discussion

This systematic review and meta-analysis aimed to quantify the prevalence of depressive disorders in childhood from 2004 to 2019, thus providing an update to the evidence previously considered by Costello et al.^31^ For depressive disorders overall (excluding DMDD), we obtained a prevalence estimate of 1.09%, which was considerably smaller than the estimate of 2.8% provided by Costello et al.^31^ Given previously discussed research suggesting that modern changes in childhood may predispose more children to depression, this might be surprising. However, there are a few potential methodologic reasons for our estimate difference; our analyses involved studies with larger sample sizes, and our ALL analyses were restricted to studies that specified an overall prevalence estimate, rather than combining results for separate disorders as performed by Costello et al.^31^ We contend that these attributes are likely to enhance the accuracy of our estimate and its stability as evinced by its small prediction interval and the results of leave-1-out sensitivity analyses increase our confidence in our results.

Additionally, we obtained specific prevalence estimates for individual depressive disorders, with rates of 0.71% for MDD, 0.30% for DYS, and 1.60% for DMDD. Disruptive mood dysregulation disorder is a relatively new diagnosis, and this likely contributed to the considerably fewer included studies. This was also compounded by studies with slightly smaller sample sizes and/or multiple cohorts, potentially limiting its applicability to other samples or geographic regions. We therefore view this figure more cautiously and would encourage further epidemiologic studies to estimate prevalence rates in a wider variety of contexts. The other 2 meta-analyses showed similar stability to the ALL analyses, with the MDD analysis particularly benefiting from a large sample size.

No appreciable differences in depressive disorder prevalence were observed between males and females. The results suggest that childhood factors may not be as salient as other potential processes leading to differences in mental health between males and females, including depression, later in life. These could include differences in the biological effects of puberty,^22^ social factors in adolescence,^72^ or increased rates of sexual violence.^73^ Nevertheless, comparatively fewer studies offered separate sex estimates of depression prevalence, so further population-wide studies sensitive to this distinction would be warranted. The sex prevalence rates for depressive disorders overall were smaller than the overall estimates, perhaps reflecting its greater sensitivity to idiosyncrasies of individual studies given the lower number of estimates that were pooled.

Like Costello et al,^31^ we found no evidence that depression has increased over time. Indeed, the results of the meta-regressions suggested that a child’s birth year, which for our models largely consisted of children born in the 1990s and 2000s, had no association with prevalence rates. The conjecture made by Costello and colleagues about historical rectification of underdiagnosis could well be applicable here too. In any case, our meta-regressions suggest that the prevalence increases observed in anxiety before the COVID-19 pandemic^24^ did not also translate to increases in depression. There could be a few reasons for this. First, given the low prevalence rates of depression, greater uncertainty around the precision of depression estimates could make it harder to detect true increases. Alternatively, increases in depression prevalence may be more easily detected when following up children over multiple sessions. Finally, although anxiety and depressive disorders share several core features (ie, negative affect and avoidant behaviors), it is possible that the causal factors leading to the increase in anxiety disorders may be unique to the nonoverlapping components of anxiety (eg, physiologic hyperarousal).

Some other noteworthy results from the meta-regressions were that older children (at least for MDD) had greater depressive prevalence rates, which would be expected as children become closer to puberty/adolescence. Heterogeneity in disorder categories may have attenuated any association that age had in the ALL analysis. For the DYS analysis, where the association with age was nonsignificant, a greater prevalence was associated with shorter interview time frames. However, given the greater risk of bias identified in the DYS studies with a shorter time frame, this finding was likely to be spurious.

One key consideration for future research is the association between the COVID-19 pandemic and the prevalence of depression in children. There is a growing body of evidence that the disruption to children’s lives due to lockdowns, quarantine, and grief have had a negative impact on their mental health.^74,75^ Additionally, in many cases, the pandemic has also worsened preexisting problems that increase the risk of depression in childhood, such as domestic violence.^76^ While our focus was on prevalence studies from 2004 to 2019, none of the COVID-19-era studies emerging from our search met all of our other inclusion criteria. This likely reflects the status of the evidence rather than a methodologic issue, although our requirement for a diagnosis to be provided by a standardized diagnostic interview could potentially have been responsible for the lack of identified studies. Previous reviews of mental health outcomes during the COVID-19 pandemic^75^ have focused on questionnaire measures, which is understandable given that conducting interviews in the context of pandemic restrictions would pose clear logistic concerns. Nevertheless, given their greater diagnostic reliability, it is important for future epidemiologic research to make use of these standardized interviews to obtain a more clinically accurate estimate of depressive disorder prevalence in children since the advent of COVID-19.

### Limitations

There were several limitations in this systematic review and meta-analysis. First, as previously discussed, we were unable to obtain a stable estimate for DMDD due to a lack of studies. Second, the current relevance of our estimates is unclear given the effect the COVID-19 pandemic has had on children’s mental health. Third, some studies were likely missed due to a few methodologic constraints placed on this meta-analysis. For example, limiting our search to reports published in English may have resulted in some missing studies. This is particularly apparent for the gray literature search, where national government–affiliated agencies from non-Anglophonic countries with potentially relevant data are numerous. Additionally, a few studies that were otherwise relevant were excluded from our analyses because they only reported prevalence estimates for cohorts containing both children and adolescents, but we did not attempt to contact any of these authors to obtain the data we needed. Finally, there was likely some level of imprecision in the meta-regression estimates, as many of the studies only reported or inferred ranges for both age and birth year, and all covariate values had to use the midpoint of the range for consistency.

## Conclusions

The findings of our meta-analyses suggest that depressive disorders in children younger than 13 years are uncommon, perhaps even more uncommon than previous estimates. Despite the increased risk posed by lifestyle factors in modern times, depressive disorders do not appear to be increasing for children younger than 13 years. The association between the COVID-19 pandemic and diagnostic prevalence is yet to be determined, but data from studies identifying depressive symptoms using questionnaires suggest that this has increased.^75^ Therefore, depression prevalence studies in childhood using standardized interviews are a research priority, as are more nuanced investigations of moderators of these outcomes.

## Notes

Supplement 1. **eTable 1.** Search Terms for the 5 Database Searches **eTable 2.** Organizations Identified From the Grey Literature Search **eMethods.** Further Methods Details **eResults.** Further Results Details **eTable 3.** Bias Assessments According to the JBIC **eTable 4.** Results of the GRADE Assessments
eReferences

Supplement 2.
Data Sharing Statement
