# Supplementary material for: Estimated Prevalence of Depressive Disorders in Children From 2004 to 2019: A Systematic Review and Meta-Analysis
Source: JAMA Pediatr. 2023 Aug 28;177(10):1017–27. doi: 10.1001/jamapediatrics.2023.3221 (PMC10463172; doi:10.1001/jamapediatrics.2023.3221)
Supplement: Supplement 1. — eTable 1. Search Terms for the 5 Database Searches eTable 2. Organizations Identified From the Grey Literature Search eMethods. Further Methods Details eResults. Further Results Details eTable 3. Bias Assessments According to the JBIC eTable 4. Results of the GRADE Assessments eReferences [file jamapediatr-e233221-s001.pdf]

# Supplementary Online Content

Spoelma MJ, Sicouri GL, Francis DA, Songco AD, Daniel EK, Hudson JL. Estimated prevalence of depressive disorders in children from 2004 to 2019: a systematic review meta-analysis. *JAMA Pediatr*. Published online August 28, 2023. doi:10.1001/jamapediatrics.2023.3221

**eTable 1.** Search Terms for the 5 Database Searches

**eTable 2.** Organizations Identified From the Grey Literature Search

**eMethods.** Further Methods Details

**eResults.** Further Results Details

**eTable 3.** Bias Assessments According to the JBIC

**eTable 4.** Results of the GRADE Assessments

**eReferences**

This supplementary material has been provided by the authors to give readers additional information about their work.

© 2023 Spoelma MJ et al. *JAMA Pediatrics*.

**eTable 1. Search terms for the 5 database searches.**

| | |
|---|---|
| | **MEDLINE (via Ovid)** |
| 1. | mental disorders/ep |
| 2. | (depress* OR mood OR affective).ti,ab |
| 3. | 1 AND 2 |
| 4. | mood disorders/ep OR depressive disorder/ep OR depressive disorder, major/ep OR depressive disorder, treatment-resistant/ep OR dysthymic disorder/ep OR cyclothymic disorder/ep |
| 5. | 3 OR 4 |
| 6. | mental disorders/ |
| 7. | 2 AND 6 |
| 8. | mood disorders/ OR depressive disorder/ OR depressive disorder, major/ OR depressive disorder, treatment-resistant/ OR dysthymic disorder/ OR cyclothymic disorder/ |
| 9. | 7 OR 8 |
| 10. | exp prevalence/ OR epidemiology/ |
| 11. | 9 AND 10 |
| 12. | 5 OR 11 |
| 13. | exp child/ OR exp child psychiatry/ OR exp "psychology, child"/ OR pediatrics/ |
| 14. | 12 AND 13 |
| 15. | (major depress* OR depressive disorder* OR dysthym* OR disruptive mood dysregulation OR MDD OR PDD OR DMDD).ti,ab |
| 16. | (prevalen* OR epidemiolog*).ti,ab |
| 17. | (child OR child* OR p?ediatr* OR pre?pube* OR primary school OR elementary school).ti,ab |
| 18. | 15 AND 16 AND 17 |
| 19. | 14 OR 18 |
| 20. | limit 19 to yr="2004-current" |
| | **PsycINFO (via Ovid)** |
| 1. | mental disorders/ |
| 2. | (depress* OR mood OR affective).ti,ab |
| 3. | 1 AND 2 |
| 4. | affective disorders/ OR major depression/ OR dysthymic disorder/ OR endogenous depression/ OR reactive depression/ OR recurrent depression/ OR treatment resistant depression/ |
| 5. | 3 OR 4 |
| 6. | epidemiology/ OR morbidity/ |
| 7. | exp child psychiatry/ OR exp child psychology/ OR exp child psychopathology/ OR exp pediatrics/ |
| 8. | (child OR children).ti,ab |
| 9. | 7 OR 8 |
| 10. | 3 AND 6 AND 9 |
| 11. | (major depress* OR depressive disorder* OR dysthym* OR disruptive mood dysregulation OR MDD OR PDD OR DMDD).ti,ab |
| 12. | (prevalen* OR epidemiolog*).ti,ab |
| 13. | (child OR child* OR p?ediatr* OR pre?pube* OR primary school OR elementary school).ti,ab |
| 14. | 11 AND 12 AND 13 |
| 15. | 10 OR 14 |
| 16. | limit 15 to yr="2004-current" |
| | **Embase (via Ovid)** |
| 1. | mental disease/ep |
| 2. | (depress* OR mood OR affective).ti,ab |
| 3. | 1 AND 2 |
| 4. | depression/ep OR agitated depression/ep OR atypical depression/ep OR chronic depression/ep OR depressive psychosis/ep OR exp dysthymia/ep OR endogenous depression/ep OR major depression/ep OR melancholia/ep OR minor depression/ep OR "mixed anxiety and depression"/ep OR organic depression/ep OR reactive depression/ep OR recurrent brief depression/ep OR treatment resistant depression/ep |
| 5. | 3 OR 4 |
| 6. | mental disease/ |

© 2023 Spoelma MJ et al. *JAMA Pediatrics*.

| | |
|---|---|
| 7. | 2 AND 6 |
| 8. | depression/ OR agitated depression/ OR atypical depression/ OR chronic depression/ OR depressive psychosis/ OR exp dysthymia/ OR endogenous depression/ OR major depression/ OR melancholia/ OR minor depression/ OR "mixed anxiety and depression"/ OR organic depression/ OR reactive depression/ OR recurrent brief depression/ OR treatment resistant depression/ |
| 9. | 7 OR 8 |
| 10. | exp prevalence/ OR epidemiology/ |
| 11. | 9 AND 10 |
| 12. | 5 OR 11 |
| 13. | child/ OR boy/ OR girl/ OR preschool child/ OR school child/ OR child psychiatry/ OR child psychology/ OR pediatrics/ |
| 14. | 12 AND 13 |
| 15. | (major depress* OR depressive disorder* OR dysthym* OR disruptive mood dysregulation OR MDD OR PDD OR DMDD).ti,ab |
| 16. | (prevalen* OR epidemiolog*).ti,ab |
| 17. | (child OR child* OR p?ediatr* OR pre?pube* OR primary school OR elementary school).ti,ab |
| 18. | 15 AND 16 AND 17 |
| 19. | 14 OR 18 |
| 20. | limit 19 to yr="2004-current" |
| **Scopus** | |
| 1. | TITLE-ABS-KEY("major depress*" OR "depressive disorder*" OR dysthym* OR "disruptive mood dysregulation" OR MDD OR PDD OR DMDD) |
| 2. | TITLE-ABS-KEY(prevalen* OR epidemiolog*) |
| 3. | TITLE-ABS-KEY(child OR child* OR p?ediatr* OR pre?pube* OR "primary school" OR "elementary school") |
| 4. | 1 AND 2 AND 3 |
| 5. | Date range: 2004-current |
| **Web of Science** | |
| 1. | (TI=(major depress* OR depressive disorder* OR dysthym* OR disruptive mood dysregulation OR MDD OR PDD OR DMDD) OR AB=(major depress* OR depressive disorder* OR dysthym* OR disruptive mood dysregulation OR MDD OR PDD OR DMDD)) AND (TI=(prevalen* OR epidemiolog*) OR AB=(prevalen* OR epidemiolog*)) AND (TI=(child OR child* OR p?ediatr* OR pre?pube* OR primary school OR elementary school) OR AB=(child OR child* OR p?ediatr* OR pre?pube* OR primary school OR elementary school)) |
| 2. | Publication date: 2004-01-01 to 3000-12-31 |

© 2023 Spoelma MJ et al. *JAMA Pediatrics*.

## eTable 2. Organizations identified from the grey literature search.

| Source/ Country | Organization |
|---|---|
| International | • World Health Organization<br>• United Nations |
| Australia | • ABS (Australian Bureau of Statistics)<br>• Australian Government Department of Health and Aged Care<br>  ○ AIHW (Australian Institute of Health and Welfare)<br>  ○ National Mental Health Commission<br>  ○ NHMRC (National Health and Medical Research Council)<br>  ○ Trove (government web archive) |
| New Zealand | • Stats NZ<br>• Ministry of Health NZ |
| UK | • Office for National Statistics<br>• Department of Health and Social Care<br>  ○ NHS (National Health Service)<br>  ○ NHS Digital |
| USA | • United States Census Bureau<br>• Department of Health and Health Services<br>  ○ AHRQ (Agency for Healthcare Research and Quality)<br>  ○ CDC (Centers for Disease Control and Prevention)<br>  ○ NIH (National Institutes of Health)<br>  ○ SAMHSA (Substance Abuse and Mental Health Services Administration) |
| Canada | • Statistics Canada<br>• Government (including Health Portfolio and three agencies below)<br>  ○ Health Canada<br>  ○ Canadian Institutes of Health Research<br>  ○ Public Health Agency of Canada |
| Ireland | • Central Statistics Office<br>• Department of Health<br>  ○ Health Research Board<br>  ○ Mental Health Commission |
| Google<br>(not specified above) | • Victoria Department of Health (Australia)<br>• Orygen<br>• OECD<br>• Mayo Clinic<br>• Australian Institute for Family Studies<br>• Mental Health Foundation UK<br>• Black Dog Institute<br>• Stanford Children's Hospital<br>• ADAA (Anxiety and Depression Association of America)<br>• RCPCH (The Royal College of Paediatrics and Child Health)<br>• Children's Hospital of Philadelphia<br>• AACAP (American Academy of Child and Adolescent Psychiatry)<br>• Victorian State Government (Australia)<br>• Telethon Kids Institute<br>• Health Direct (Australia)<br>• Beyond Blue<br>• NICE (The National Institute for Health and Care Excellence)<br>• Cleveland Clinic<br>• Caring for Kids New to Canada |

© 2023 Spoelma MJ et al. *JAMA Pediatrics*.

## eMethods. Further Methods Details

All procedures outlined below and in the main manuscript were consistent with the MOOSE guidelines.[1] The analyses underlying this paper were originally commissioned for an institutional report produced by the Black Dog Institute, and such an arrangement meant that this review was not pre-registered.

### Search Strategy and Study Selection

The grey literature search detailed in the Methods and eTable 2 was conducted according to a systematic strategy previously outlined by Godin et al.[2]

The seventh inclusion criterion stated in the main text is that data collection must have started no earlier than 2002 and ended no later than 2019. The choice to the include this criterion was made to ensure that the estimates we obtained were truly reflective of our specified time period of 2004-2019. To illustrate this concern, we highlight one study by McArdle et al.[3] which was identified by our search and met all our inclusion criteria aside from the seventh. Despite being published in 2004, this study evaluates prevalence estimates from a decade earlier.

This could potentially be a problem, particularly for studies on the older side of our date range. However, we also note that it is reasonable to expect some delay between the end of data collection and study publication, and that prevalence estimates which are only a few years old are likely to still be applicable in 2004. Additionally, we note that the previous meta-analysis by Costello et al.[4] that we are building upon considered studies that were published no later than 2004 and had finished data collection no later than 2001. We therefore believe this justifies a data collection end date lower limit of 2002.

The title/abstract and full-text screening showed good inter-rater reliability (agreement 99.50%, $\kappa = 0.695$) (agreement 94.29%, $\kappa = 0.870$).

### Data Extraction

MJS and JLH completed data extraction independently. Initial agreement was good (94.90%), but all discrepancies were resolved by discussion.

Only studies reporting a separate overall estimate were included in the ALL meta-analysis so that rates were not affected by comorbidity presentations (such as the phenomenon of "double depression"). Additionally, due to heterogeneity in diagnostic naming, for the purposes of our analyses, we considered our MDD category to encompass major depressive disorder, major depressive episodes, and ICD-defined moderate and severe depression. Minor depression, ICD-defined mild depression, depression not otherwise specified, and miscellaneous other depression labels were not considered for the diagnostic-specific analyses, but these were accounted for in the ALL analyses.

### Data Synthesis and Analysis

The choice of variance estimator, estimate transformation, and confidence intervals have been previously recommended by Munn et al.[5] Additionally, the choice to measure study heterogeneity using prediction intervals and leave-one-out sensitivity analyses was recommended by Migliavaca et al.,[6] who illustrated that $I^2$ (a traditional meta-analysis heterogeneity metric) is commonly inaccurate for prevalence studies.

For the subgroup analyses, low-and-middle-income and high-income countries (LMICs and HICs respectively) were defined based on definitions given by the World Bank. For our studies, the LMICs assessed were Bangladesh, Brazil, China, Iran, Türkiye, and Yemen. The HICs assessed were Australia, Canada, Chile, Denmark, England, Iceland, Netherlands, Norway, Saudi Arabia, South Korea, Spain, Taiwan, and the United States of America.

The bias assessments were conducted using the Joanna Briggs Institute Critical Appraisal Checklist for Prevalence Studies (JBIC), created by Munn et al.[5] Inter-rater reliability was strong (agreement 96.17%, $\kappa = 0.921$).

For Question 3, in line with recommendations,[5,7] an "adequate sample size" was defined as one that exceeds *n*, given by the formula below:

© 2023 Spoelma MJ et al. *JAMA Pediatrics*.

$$n = \frac{z^2 p(1-p)}{d^2}$$

Here, $z$ is the $z$-statistic for a specified level of confidence (for $\alpha = 0.05$, this was $z = 1.96$), $p$ is the expected prevalence (in the absence of any further prior information, we set $p = 0.028$ in line with the previously obtained estimate by Costello et al.[4]), and $d$ is the precision (consistent with recommendations by Naing et al.,[7] this was set as $d = 0.014 = p/2$).

Our confidence in the overall prevalence estimates (and overall study quality) was assessed using the GRADE approach. While GRADE has not been specifically tailored for proportional meta-analyses of prevalence, applying guidelines for prognostic studies[8] has been recommended as an adequate alternative.[9]

© 2023 Spoelma MJ et al. *JAMA Pediatrics*.

## eResults. Further Results Details

Slightly under half of the studies examined populations from Western (*n* = 20, 48.8%) and low-and-middle-income countries (*n* = 17, 41.5%) respectively. Most (*n* = 38, 92.7%) employed a DSM taxonomy, with only three studies[10-12] using an ICD-10 taxonomy exclusively. Seven different interview instruments were used, the most popular being the DAWBA[13] (*n* = 12, 29.3%), K-SADS[14] (*n* = 10, 24.4%), and DISC-IV[15] (*n* = 7, 17.1%), while the PAPA[16] (*n* = 5, 12.2%) was often used for children aged 6 or under. These interviews were mostly (*n* = 38, 92.7%) informed by parents, either exclusively or occasionally in addition to child or teacher input.

Prevalence rates were calculated using raw case numbers when possible. When only rates were provided for either the prevalence or the sample size of a potentially relevant subsample (e.g., children in a combined child/adolescent sample, or gender sub-groups), a raw number was estimated by multiplying the rate by the total sample size and rounding to the nearest natural number. Additionally, many studies employed a multi-staged recruitment strategy in which a screening measure, such as the Strengths and Difficulties Questionnaire,[17] was used to determine who would progress to the interview stage. Dropouts occurred for various reasons between these two stages. In these circumstances, unless otherwise specified in the papers, the sample size used for the prevalence estimates was assumed to be the number of individuals who completed the screening measure. The potential effect of dropouts on the over- or under-estimation of the prevalence rates are considered in the bias assessments.

Five studies offered separate prevalence estimates according to an impairment criterion. Impairment was either specified as being present/absent,[18,19] or of specified severity levels (i.e., mild, severe).[20-22] In this paper, prevalence rates are reported for conditions that reported at least mild impairment. A further three studies[23-25] compared depression with “subthreshold” depression. In these cases, the former estimates were used.

Excluding gender-based prevalence rates, three studies reported prevalence for multiple cohorts; all of these were defined based on age groups. Two of these[26,27] used independent samples, and were thus considered separately in the meta-analyses. However, one longitudinal study[28] followed a single cohort and reported prevalence rates every two years from the ages of 4-14. Thus, to avoid having a disproportionate influence on the results, the estimates used for Morken et al.[28] were the prevalence rates for MDD and DYS averaged over all childhood ages (i.e., 4-12).

A similar averaging approach was required for two cohorts that were examined across multiple studies at different ages. These cohorts are referred to in the forest plots as Pelotas Combined and Stony Brook Combined. The former was required to combine an MDD estimate from two studies,[29,30] while the latter combined DMDD and ALL estimates across four studies.[31-34] In some cases, two studies considered an individual cohort at the same time point but offered differing estimates. For these, the study with the greater number of participants was used. Specifically, the MDD and DYS estimates of Wichstrøm et al.[35] were superseded by Morken et al.,[28] and the DMDD estimates of Munhoz et al.[36] were superseded by La Maison et al.[29]

Only one study[29] reported a combined depressive disorder prevalence estimate that included DMDD. This was likely given its relatively new inclusion as a separate diagnosis in DSM-5. Therefore, given the lack of other studies to compare against the prevalence of DMDD in the context of depressive disorders overall, DMDD is not included in the ALL analyses and is only considered separately.

© 2023 Spoelma MJ et al. *JAMA Pediatrics*.

## eTable 3. Bias assessments according to the JBIC.

| | JBIC Bias Assessment[a] | | | | | | | | | |
|---|---|---|---|---|---|---|---|---|---|---|
| **Study** | **Q1** | **Q2** | **Q3[b]** | **Q4** | **Q5** | **Q6** | **Q7** | **Q8** | **Q9** | **Total** |
| Al-Modayfer & Alatiq,[37] 2015 | – | ✓ | X | X | – | ✓ | – | – | – | 2 |
| Alyahri & Goodman,[38] 2008 | ✓ | ✓ | ✓ | X | ✓ | ✓ | – | ✓ | ✓ | 7 |
| Amiri et al.,[39] 2019 | ✓ | ✓ | X | – | – | ✓ | – | X | – | 3 |
| Anselmi et al.,[40] 2010 | ✓ | ✓ | ✓ | ✓ | X | ✓ | ✓ | ✓ | X | 7 |
| Bufferd et al.,[31] 2011 | ✓ | ✓ | X | ✓ | ✓ | ✓ | ✓ | ✓ | X | 7 |
| Bufferd et al.,[32] 2012 | ✓ | ✓ | X | ✓ | ✓ | ✓ | ✓ | ✓ | X | 7 |
| Canals-Sans et al.,[41] 2018 | ✓ | ✓ | ✓ | ✓ | X | ✓ | – | ✓ | X | 6 |
| Carter et al.,[18] 2010 | ✓ | ✓ | ✓ | ✓ | X | ✓ | X | ✓ | X | 6 |
| Deng et al.,[42] 2023 | ✓ | ✓ | ✓ | X | – | ✓ | – | ✓ | – | 5 |
| Dodangi et al.,[43] 2014 | ✓ | ✓ | X | X | – | ✓ | X | X | – | 3 |
| Dougherty et al.,[33] 2014 | ✓ | ✓ | X | ✓ | X | ✓ | ✓ | X | X | 5 |
| Dougherty et al.,[34] 2016 | – | – | X | ✓ | X | ✓ | ✓ | X | X | 3 |
| Dursun et al.,[23] 2020 | ✓ | ✓ | ✓ | ✓ | X | ✓ | X | X | X | 5 |
| Elberling et al.,[10] 2016 | ✓ | ✓ | ✓ | ✓ | ✓ | ✓ | ✓ | ✓ | ✓ | 9 |
| Ezpeleta et al.,[44] 2014 | ✓ | ✓ | ✓ | ✓ | X | ✓ | – | ✓ | ✓ | 6 |
| Georgiades et al.,[45] 2019 | ✓ | ✓ | ✓ | ✓ | X | ✓ | – | X | X | 5 |
| Gudmundsson et al.,[46] 2013 | ✓ | ✓ | X | ✓ | X | ✓ | – | X | X | 4 |
| Heiervang et al.,[47] 2007 | ✓ | ✓ | ✓ | – | ✓ | ✓ | ✓ | ✓ | ✓ | 8 |
| Karacetin et al.,[19] 2018 | ✓ | ✓ | ✓ | – | – | ✓ | X | X | X | 4 |
| La Maison et al.,[29] 2018 | ✓ | ✓ | ✓ | ✓ | ✓ | ✓ | ✓ | ✓ | ✓ | 9 |
| Lavigne et al.,[20] 2009 | ✓ | ✓ | ✓ | ✓ | X | ✓ | X | ✓ | X | 6 |
| Lawrence et al.,[48] 2015 | ✓ | ✓ | ✓ | – | ✓ | ✓ | – | ✓ | – | 6 |
| Lin et al.,[26] 2021 | ✓ | ✓ | ✓ | – | ✓ | ✓ | – | ✓ | ✓ | 7 |
| Merikangas et al.,[21] 2010 | ✓ | ✓ | ✓ | X | ✓ | ✓ | – | ✓ | X | 6 |
| Mohammadi et al.,[49] 2019 | ✓ | ✓ | ✓ | X | – | ✓ | – | X | – | 4 |
| Morken et al.,[28] 2021 | ✓ | ✓ | ✓ | ✓ | ✓ | ✓ | ✓ | ✓ | ✓ | 9 |
| Mullick & Goodman,[11] 2005 | ✓ | X | ✓ | ✓ | X | ✓ | X | X | – | 4 |
| Munhoz et al.,[36] 2017 | ✓ | ✓ | ✓ | ✓ | ✓ | ✓ | ✓ | – | ✓ | 8 |
| Olfson et al.,[50] 2023 | ✓ | ✓ | ✓ | ✓ | – | ✓ | – | X | ✓ | 6 |
| Park et al.,[24] 2014 | ✓ | ✓ | ✓ | – | ✓ | ✓ | X | ✓ | ✓ | 7 |
| Petresco et al.,[30] 2014 | ✓ | ✓ | ✓ | ✓ | ✓ | ✓ | ✓ | ✓ | ✓ | 9 |
| Rijlaarsdam et al.,[22] 2015 | ✓ | ✓ | ✓ | – | ✓ | ✓ | – | ✓ | ✓ | 7 |
| Salum et al.,[51] 2015 | ✓ | ✓ | ✓ | ✓ | X | ✓ | ✓ | X | X | 6 |
| Shen et al.,[52] 2018 | ✓ | ✓ | ✓ | X | – | ✓ | ✓ | X | – | 5 |
| Tüğen et al.,[53] 2020 | X | – | X | X | – | ✓ | – | X | X | 1 |
| Vicente et al.,[54] 2012 | ✓ | ✓ | ✓ | – | – | ✓ | – | – | ✓ | 5 |
| Vizard et al.,[12] 2018 | ✓ | ✓ | ✓ | – | – | ✓ | – | ✓ | ✓ | 6 |
| Wesselhoeft et al.,[25] 2016 | ✓ | ✓ | ✓ | X | ✓ | ✓ | ✓ | X | X | 6 |
| Wichstrøm et al.,[35] 2012 | ✓ | ✓ | ✓ | ✓ | ✓ | ✓ | ✓ | ✓ | ✓ | 9 |
| Yadegari et al.,[55] 2022 | ✓ | ✓ | X | X | X | ✓ | X | X | X | 3 |
| Zhong et al.,[27] 2013 | ✓ | ✓ | ✓ | ✓ | ✓ | ✓ | ✓ | ✓ | ✓ | 9 |

[a] X = no (0), – = unclear (0), ✓ = yes (1).
**[b]** See eResults for details on the assessment of Q3.

© 2023 Spoelma MJ et al. *JAMA Pediatrics*.

**eTable 4. Results of the GRADE assessments.**

| Quality Assessment | | | | | | Prevalence (95% CI) | Quality |
|---|---|---|---|---|---|---|---|
| **No. of Studies** | **Risk of Bias[a]** | **Inconsistency[b]** | **Indirectness[c]** | **Imprecision[d]** | **Publication Bias[e]** | | |
| **Prevalence of all depressive disorders** | | | | | | | |
| 19 | Not serious | Not serious | Not serious | Not serious | N/A | 1.06 (0.62, 1.63) | High |
| **Prevalence of major depressive disorder** | | | | | | | |
| 29 | Not serious | Not serious | Not serious | Not serious | N/A | 0.71 (0.48, 0.99) | High |
| **Prevalence of dysthymia** | | | | | | | |
| 16 | Not serious | Not serious | Not serious | Not serious | N/A | 0.30 (0.08, 0.62) | High |
| **Prevalence of disruptive mood dysregulation disorder** | | | | | | | |
| 5 | Not serious | Serious | Not serious | Serious | N/A | 1.60 (0.28, 3.90) | Low |

CI = confidence interval, N/A = not applicable.
[a] Ascertained through JBIC and sensitivity analyses. DMDD had one study with high risk of bias, but this was offset by other very high-quality studies and the fact that the estimate did not differ from them anyway.
[b] Estimates are quite similar overall. No differences in subgroups of interest. Occasional aberrant study offset by the large number of other studies (except DMDD). Traditional heterogeneity statistics not appropriate for prevalence meta-analyses.
[c] All studies involved population data and were thus by definition generalizable.
[d] Most confidence intervals are reasonable, and those that are not reasonable are offset by those that are (except DMDD).
[e] Given that prevalence measures are not usually subject to traditional tests of statistical significance, we judge that publication bias is not a salient concern.

© 2023 Spoelma MJ et al. *JAMA Pediatrics*.

**eReferences**


1. Stroup DF, Berlin JA, Morton SC, et al. Meta-analysis of observational studies in epidemiology: a proposal for reporting. *JAMA*. 2000;283(15):2008-2012. doi:10.1001/jama.283.15.2008
2. Godin K, Stapleton J, Kilpatrick SI, Hanning RM, Leatherdale ST. Applying systematic review search methods to the grey literature: a case study examining guidelines for school-based programs in Canada. *Syst Rev*. 2015;4:138. doi:10.1186/s13643-015-0125-0
3. McArdle P, Prosser J, Kolvin I. Prevalence of psychiatric disorder: with and without psychosocial impairment. Eur Child Adolesc Psychiatry. 2004;13(6):347-353. doi:10.1007/s00787-004-0367-1
4. Costello EJ, Erkanli A, Angold A. Is there an epidemic of child or adolescent depression? J Child Psychol Psychiatry. 2006;47(12):1263-1271. doi:10.1111/j.1469-7610.2006.01682.x
5. Munn Z, Moola S, Lisy K, Riitano D, Tufanaru C. Methodological guidance for systematic reviews of observational epidemiological studies reporting prevalence and cumulative incidence data. *Int J Evid Based Healthc*. 2015;13:147-153. doi:10.1097/XEB.0000000000000054
6. Migliavaca CB, Stein C, Colpani V, et al. Meta-analysis of prevalence: *I*2 statistic and how to deal with heterogeneity. *Res Synth Methods*. 2021;13:363-367. doi:10.1002/jrsm.1547
7. Naing L, Winn T, Rusli BN. Practical issues in calculating the sample size for prevalence studies. *Arch Orofac Sci*. 2006;1:9-14.
8. Iorio A, Spencer FA, Falavigna M, et al. Use of GRADE for assessment of evidence about prognosis: rating confidence in estimates of event rates in broad categories of patients. BMJ. 2015;350:h870. doi:10.1136/bmj.h870
9. Migliavaca CB, Stein C, Colpani V, Barker TH, Munn Z, Falavigna M. How are systematic reviews of prevalence conducted? A methodological study. BMC Med Res Methodol. 2020;20:96. doi:10.1186/s12874-020-00975-3
10. Elberling H, Linneberg A, Rask CU, Houman T, Goodman R, Skovgaard AM. Psychiatric disorders in Danish children aged 5-7 years: a general population study of prevalence and risk factors from the Copenhagen Child Cohort (CCC 2000). *Nord J Psychiatry*. 2016;70(2):146-155. doi:10.3109/08039488.2015.1070199
11. Mullick MSI, Goodman R. The prevalence of psychiatric disorders among 5–10 year olds in rural, urban and slum areas in Bangladesh. *Soc Psychiatry Psychiatr Epidemiol.* 2005;40(8):663-671. doi:10.1007/s00127-005-0939-5
12. Vizard T, Pearce N, Davis J, et al. Mental Health of Children and Young People in England, 2017. NHS Digital. November 22, 2018. Accessed November 11, 2022. https://digital.nhs.uk/data-and-information/publications/statistical/mental-health-of-children-and-young-people-in-england/2017/2017
13. Goodman R, Ford T, Richards H, Gatward R, Meltzer H. The Development and Well-Being Assessment: description and initial validation of an integrated assessment of child and adolescent psychopathology. *J Child Psychol Psychiatry*. 2000;41(5):645-655. doi:10.1111/j.1469-7610.2000.tb02345.x
14. Kaufman J, Birmaher B, Brent D, et al. Schedule for Affective Disorders and Schizophrenia for School-Age Children-Present and Lifetime Version (K-SADS-PL): initial reliability and validity data. *J Am Acad Child Adolesc Psychiatry*. 1997;36(7):980-988. doi:10.1097/00004583-199707000-00021
15. Shaffer D, Fisher P, Lucas CP, Dulcan MK, Schwab-Stone ME. NIMH Diagnostic Interview Schedule for Children Version IV (NIMH DISC-IV): description, differences from previous versions, and reliability of some common diagnoses. *J Am Acad Child Adolesc Psychiatry*. 2000;39(1):28-38. doi:10.1097/00004583-200001000-00014
16. Egger HL, Erkanli A, Keeler G, Potts E, Walter BK, Angold A. Test-retest reliability of the Preschool Age Psychiatric Assessment (PAPA). *J Am Acad Child Adolesc Psychiatry*. 2006;45(5):538-549. doi:10.1097/01.chi.0000205705.71194.b8
17. Goodman R. The strengths and difficulties questionnaire: a research note. *J Child Psychol Psychiatry*. 1997;38(5):581-586. doi:10.1111/j.1469-7610.1997.tb01545.x
18. Carter AS, Wagmiller RJ, Gray SAO, McCarthy KJ, Horwitz SM, Briggs-Gowan MJ. Prevalence of DSM-IV disorder in a representative, healthy birth cohort at school entry: sociodemographic risks and social adaptation. *J Am Acad Child Adolesc Psychiatry*. 2010;49(7):686-698. doi:10.1016/j.jaac.2010.03.018
19. Karacetin G, Arman AR, Fis NP, et al. Prevalence of childhood affective disorders in Turkey: an epidemiological study. *J Affect Disord*. 2018;238:513-521. doi:10.1016/j.jad.2018.05.014
20. Lavigne JV, LeBailly SA, Hopkins J, Gouze KR, Binns HJ. The prevalence of ADHD, ODD, depression, and anxiety in a community sample of 4-year-olds. *J Clin Child Adolesc Psychol*. 2009;38(3):315-328. doi:10.1080/15374410902851382
21. Merikangas KR, He J-P, Brody D, Fisher PW, Bourdon K, Koretz DS. Prevalence and treatment of mental disorders among US children in the 2001-2004 NHANES. *Pediatrics*. 2010;125(1):75-81. doi:10.1542/peds.2008-2598


© 2023 Spoelma MJ et al. *JAMA Pediatrics*.

22. Rijlaarsdam J, Stevens GWJM, van der Ende J, et al. Prevalence of DSM-IV disorders in a population-based sample of 5- to 8-year-old children: the impact of impairment criteria. *Eur Child Adolesc Psychiatry*. 2015;24:1339-1348. doi:10.1007/s00787-015-0684-6
23. Dursun OB, Esin İS, Akıncı MA, Karayağmurlu A, Turan B, Aşıkhasanoğlu EÖ. The prevalence of childhood mental disorders in different habitations: are we underestimating their prevalence in rural areas? *Nord J Psychiatry*. 2020;74(3):201-207. doi:10.1080/08039488.2019.1689295
24. Park S, Kim B-N, Cho S-C, Kim J-W, Shin M-S, Yoo H-J. Prevalence, correlates, and comorbidities of DSM-IV psychiatric disorders in children in Seoul, Korea. *Asia Pac J Public Health*. 2014;27(2):NP1942-NP1951. doi:10.1177/1010539513475656
25. Wesselhoeft R, Heiervang ER, Kragh-Sorensen P, Juul Sorensen M, Bilenberg N. Major depressive disorder and subthreshold depression in prepubertal children from the Danish National Birth Cohort. *Compr Psychiatry*. 2016;70:65-76. doi:10.1016/j.comppsych.2016.06.012
26. Lin Y-J, Tseng W-L, Gau SS-F. Psychiatric comorbidity and social adjustment difficulties in children with disruptive mood dysregulation disorder: a national epidemiological study. *J Affect Disord*. 2021;281:485-492. doi:10.1016/j.jad.2020.12.039
27. Zhong B-L, Ding J, Chen H-H, et al. Depressive disorders among children in the transforming China: an epidemiological survey of prevalence, correlates, and service use. *Depress Anxiety*. 2013;30(9):881-892. doi:10.1002/da.22109
28. Morken IS, Viddal KR, Ranum B, Wichstrøm L. Depression from preschool to adolescence - five faces of stability. *J Child Psychol Psychiatry*. 2021;62(8):1000-1009. doi:10.1111/jcpp.13362
29. La Maison C, Munhoz TN, Santos IS, Anselmi L, Barros FC, Matijasevich A. Prevalence and risk factors of psychiatric disorders in early adolescence: 2004 Pelotas (Brazil) birth cohort. *Soc Psychiatry Psychiatr Epidemiol*. 2018;53(7):685-697. doi:10.1007/s00127-018-1516-z
30. Petresco S, Anselmi L, Santos IS, et al. Prevalence and comorbidity of psychiatric disorders among 6-year-old children: 2004 Pelotas Birth Cohort. *Soc Psychiatry Psychiatr Epidemiol*. 2014;49(6):975-983. doi:10.1007/s00127-014-0826-z
31. Bufferd SJ, Dougherty LR, Carlson GA, Klein DN. Parent-reported mental health in preschoolers: findings using a diagnostic interview. *Compr Psychiatry*. 2011;52(4):359-369. doi:10.1016/j.comppsych.2010.08.006
32. Bufferd SJ, Dougherty LR, Carlson GA, Rose S, Klein DN. Psychiatric disorders in preschoolers: continuity from ages 3 to 6. *Am J Psychiatry*. 2012;169(11):1157-1164. doi:10.1176/appi.ajp.2012.12020268
33. Dougherty LR, Smith VC, Bufferd SJ, et al. DSM-5 disruptive mood dysregulation disorder: correlates and predictors in young children. *Psychol Med*. 2014;44(11):2339-2350. doi:10.1017/S0033291713003115
34. Dougherty LR, Smith VC, Bufferd SJ, Kessel EM, Carlson GA, Klein DN. Disruptive mood dysregulation disorder at the age of 6 years and clinical and functional outcomes 3 years later. *Psychol Med*. 2016;46(5):1103-1114. doi:10.1017/S0033291715002809
35. Wichstrøm L, Berg-Nielsen TS, Angold A, Egger HL, Solheim E, Sveen TH. Prevalence of psychiatric disorders in preschoolers. *J Child Psychol Psychiatry*. 2012;53(6):695-705. doi:10.1111/j.1469-7610.2011.02514.x
36. Munhoz TN, Santos IS, Barros AJD, Anselmi L, Barros FC, Matijasevich A. Perinatal and postnatal risk factors for disruptive mood dysregulation disorder at age 11: 2004 Pelotas Birth Cohort Study. *J Affect Disord*. 2017;215:263-268. doi:10.1016/j.jad.2017.03.040
37. Al-Modayfer O, Alatiq Y. A pilot study on the prevalence of psychiatric disorders among Saudi children and adolescents: a sample from a selected community in Riyadh city. *Arab J Psychiatry*. 2015;26(2):184-192. doi:10.12816/0014485
38. Alyahri A, Goodman R. The prevalence of DSM-IV psychiatric disorders among 7–10 year old Yemeni schoolchildren. *Soc Psychiatry Psychiatr Epidemiol*. 2008;43(3):224-230. doi:10.1007/s00127-007-0293-x
39. Amiri S, Mohammadi MR, Ahmadi N, et al. Prevalence of psychiatric disorders among children and adolescents in the East Azerbaijan province, Tabriz, Iran, in 2017. *Iran J Psychiatry Behav Sci*. 2019;13(4):e88594. doi:10.5812/ijpbs.88594
40. Anselmi L, Fleitlich-Bilyk B, Menezes AMB, Araújo CL, Rohde LA. Prevalence of psychiatric disorders in a Brazilian birth cohort of 11-year-olds. *Soc Psychiatry Psychiatr Epidemiol*. 2010;45(1):135-142. doi:10.1007/s00127-009-0052-2
41. Canals-Sans J, Hernández-Martínez C, Sáez-Carles M, Arija-Val V. Prevalence of DSM-5 depressive disorders and comorbidity in Spanish early adolescents: has there been an increase in the last 20 years? *Psychiatry Res*. 2018;268:328-334. doi:10.1016/j.psychres.2018.07.023
42. Deng H, Wen F, Xu H, et al. Prevalence of affective disorders in Chinese school-attending children and adolescents aged 6-16 based on a national survey by MINI-Kid. *J Affect Disord*. 2023;331:192-199. doi:10.1016/j.jad.2023.03.060

© 2023 Spoelma MJ et al. *JAMA Pediatrics*.

43. Dodangi N, Ashtiani NH, Valadbeigi B. Prevalence of DSM-IV TR psychiatric disorders in children and adolescents of Paveh, a western city of Iran. *Iran Red Crescent Med J*. 2014;16(7):e16743. doi:10.5812/ircmj.16743
44. Ezpeleta L, de la Osa N, Doménech JM. Prevalence of DSM-IV disorders, comorbidity and impairment in 3-year-old Spanish preschoolers. *Soc Psychiatry Psychiatr Epidemiol*. 2014;49(1):145-155. doi:10.1007/s00127-013-0683-1
45. Georgiades K, Duncan L, Wang L, Comeau J, Boyle MH. Six-month prevalence of mental disorders and service contacts among children and youth in Ontario: evidence from the 2014 Ontario Child Health Study. *Can J Psychiatry*. 2019;64(4):246-255. doi:10.1177/0706743719830024
46. Gudmundsson OO, Magnusson P, Saemundsen E, et al. Psychiatric disorders in an urban sample of preschool children. *Child Adolesc Ment Health*. 2013;18(4):210-217. doi:10.1111/j.1475-3588.2012.00675.x
47. Heiervang E, Stormark KM, Lundervold AJ, et al. Psychiatric disorders in Norwegian 8- to 10-year-olds: an epidemiological survey of prevalence, risk factors, and service use. *J Am Acad Child Adolesc Psychiatry*. 2007;46(4):438-447. doi:10.1097/chi.0b013e31803062bf
48. Lawrence D, Johnson S, Hafekost J, et al. The mental health of children and adolescents: report on the second Australian child and adolescent survey of mental health and wellbeing. Commonwealth of Australia. August, 2015. Accessed November 15, 2022. https://www.health.gov.au/resources/publications/the-mental-health-of-children-and-adolescents.
49. Mohammadi MR, Alavi SS, Ahmadi N, et al. The prevalence, comorbidity and socio-demographic factors of depressive disorder among Iranian children and adolescents: to identify the main predictors of depression. *J Affect Disord*. 2019;247:1-10. doi:10.1016/j.jad.2019.01.005
50. Olfson M, Wall MM, Wang S, Blanco C. Prevalence and correlates of mental disorders in children aged 9 and 10 years: results from the ABCD study. *J Am Acad Child Adolesc Psychiatry*. 2023. doi:10.1016/j.jaac.2023.04.005
51. Salum GA, Gadelha A, Pan PM, et al. High risk cohort study for psychiatric disorders in childhood: rationale, design, methods and preliminary results. *Int J Methods Psychiatr Res*. 2015;24(1):58-73. doi:10.1002/mpr.1459
52. Shen Y-M, Chan BSM, Liu J-B, et al. The prevalence of psychiatric disorders among students aged 6~ 16 years old in central Hunan, China. *BMC Psychiatry*. 2018;18(1):243. doi:10.1186/s12888-018-1823-7
53. Tüğen LE, Göksu M, Ayaz AB. Disruptive mood dysregulation disorder in a primary school sample. *Asian J Psychiatry*. 2020;48:101858. doi:10.1016/j.ajp.2019.101858
54. Vicente B, Saldivia S, de la Barra F, et al. Prevalence of child and adolescent mental disorders in Chile: a community epidemiological study. *J Child Psychol Psychiatry*. 2012;53(10):1026-1035. doi:10.1111/j.1469-7610.2012.02566.x
55. Yadegari N, Jouybari L, Fathi M, et al. Epidemiology of psychiatric disorders among children and adolescents in Kohgiloyeh and Boyer- Ahmad province, Iran. *J Adv Med Biomed Res*. 2022;30(141):365-373. doi:10.30699/jambs.30.141.365


© 2023 Spoelma MJ et al. *JAMA Pediatrics*.
