# Supplementary material for: Estimated Prevalence of Depressive Disorders in Children From 2004 to 2019: A Systematic Review and Meta-Analysis
Source: JAMA Pediatr. 2023 Aug 28;177(10):1017–27. doi: 10.1001/jamapediatrics.2023.3221 (PMC10463172; doi:10.1001/jamapediatrics.2023.3221)
Supplement: Supplement 2. — Data Sharing Statement [file jamapediatr-e233221-s002.pdf]

Data Sharing Statement

Spoelma. Estimated Prevalence of Depressive Disorders in Children From 2004 to 2019. *JAMA Pediatr*. Published August 28, 2023. doi:10.1001/jamapediatrics.2023.3221

Data
Data available: No

Additional Information
Explanation for why data not available: All data are available in the paper and in the published literature.
